# Combination of Enzymes with Materials to Give Them Antimicrobial Features: Modern Trends and Perspectives

**DOI:** 10.3390/jfb14020064

**Published:** 2023-01-25

**Authors:** Elena Efremenko, Nikolay Stepanov, Aysel Aslanli, Ilya Lyagin, Olga Senko, Olga Maslova

**Affiliations:** 1 Faculty of Chemistry, Lomonosov Moscow State University, Lenin Hills 1/3, 119991 Moscow, Russia; 2 N.M. Emanuel Institute of Biochemical Physics RAS, Kosygin str. 4, 119334 Moscow, Russia

**Keywords:** oxidoreductases, hydrolyses, lactonases, antibiotics, antimicrobial peptides, metal nanoparticles, computational modeling, immobilization, functionalized materials, biocompatibility

## Abstract

Multidrug-resistant bacteria form serious problems in many areas, including medicine and the food industry. At the same time, great interest is shown in the transfer or enhancement of antimicrobial properties to various materials by modifying them with enzymes. The use of enzymes in biomaterials with antimicrobial properties is important because enzymes can be used as the main active components providing antimicrobial properties of functionalized composite biomaterials, or can serve as enhancers of the antimicrobial action of certain substances (antibiotics, antimicrobial peptides, metal nanoparticles, etc.) against cells of various microorganisms. Enzymes can simultaneously widen the spectrum of antimicrobial activity of biomaterials. This review presents the most promising enzymes recently used for the production of antibacterial materials, namely hydrolases and oxidoreductases. Computer modeling plays an important role in finding the most effective combinations between enzymes and antimicrobial compounds, revealing their possible interactions. The range of materials that can be functionalized using enzymes looks diverse. The physicochemical characteristics and functionalization methods of the materials have a significant impact on the activity of enzymes. In this context, fibrous materials are of particular interest. The purpose of this review is to analyze the current state of the art in this area.

## 1. Introduction

Recently, there has been a growing interest in antimicrobial materials, especially in various fields of human action, especially in human and veterinary medicine, sanitary, food industry, etc. [1,2]. This is due to the loss of the effectiveness of many blockbuster antibiotics and antimicrobials used widely today, as well as the development of the global problem of antibiotic resistance [3]. 

The search or synthesis of new antibiotics is a long and costly process. In this regard, it is relevant to find new antimicrobial drugs/materials with different mechanisms of action and targets of exposure individually or in effective combinations [4]. Bacterial adhesion to materials strongly depends on their physicochemical properties. One of the approaches used in the development of antibacterial coatings is oriented on the weakening of adhesion of microbial cells on materials by changing their surface properties [5]. Another approach is based on the materials’ treatment with active biocides. Recently, the approach using multiple functionalizations for obtaining antibacterial materials via a combination of various antimicrobial agents has been investigated [6,7]. Broad-spectrum antimicrobials capable of preventing the bacterial contamination of materials with low cytotoxicity and stability for a long time were in the center of the growing interest.

One of the current trends in the development of a strategy for combating bacterial contaminations and creating combined antimicrobial materials is focused on the use of different enzymes [8,9,10]. To obtain antibacterial materials, enzymes are used both individually and in combination with other enzymes, antimicrobial peptides, antibiotics, metal nanoparticles, etc. As a rule, the use of enzymes involves their stabilization by various methods. Initial enzymes can be modified using classical methods of rational design and directed evolution to introduce additional salt bridges, hydrogen bonds, disulfide bonds, electrostatically and/or hydrophobically interacting groups, etc. [11]. Application of advanced computational methods, including machine learning models [12], is the most effective to pre-select sites for such modification. Further, enzymes can form complexes or be conjugated with various inorganic and organic stabilizers, including nanomaterials [13] and polymers [14]. Moreover, partners for the complexes can be also rationally selected using computer modeling to minimally affect the natural confirmation of the enzyme molecule, and thus, to prevent activity losses. Various carriers can be used to immobilize stabilized forms of enzymes and anti-microbial combinations by well-known immobilizing techniques (sorption, encapsulation, chemical, and affine binding [15]), providing functional biomaterials. 

The aim of this review was to conduct a sampling and analysis of studies carried out over the past 5 years focused on the practical application of enzymes for the production of antibacterial materials. The articles were searched in several scientific databases [https://scholar.google.com (accessed on 20 December 2022), https://pubmed.ncbi.nlm.nih.gov (accessed on 20 December 2022)] by using combinations of following keywords: antimicrobial materials, enzymes, oxidoreductases, hydrolyses, lactonases, antimicrobial peptides, antibiotics, antibiotic resistance, metal-organic frameworks. At the same time, there was no task to identify all possible options for existing solutions, but it was interesting, from a scientific and practical point of view, to determine the main current trends in the developments of antimicrobial materials containing enzymes. A Google Scholar search gave the highest number of documents (~17,700 documents), and it was followed by a PubMed (1,539 documents) due to its narrower scope. The information that was selected for analysis included different classes of enzymes with their typical catalytic activity, an assessment of their possible interactions with various antimicrobial drugs, the effectiveness of which was confirmed, among other things, by the results of computer modeling.

## 2. Enzymes in Combinations with Other Enzymes, Antibiotics, Nanoparticles and Antimicrobial Peptides in the Content of Various Materials with Antimicrobial Properties

**Figure 1:**
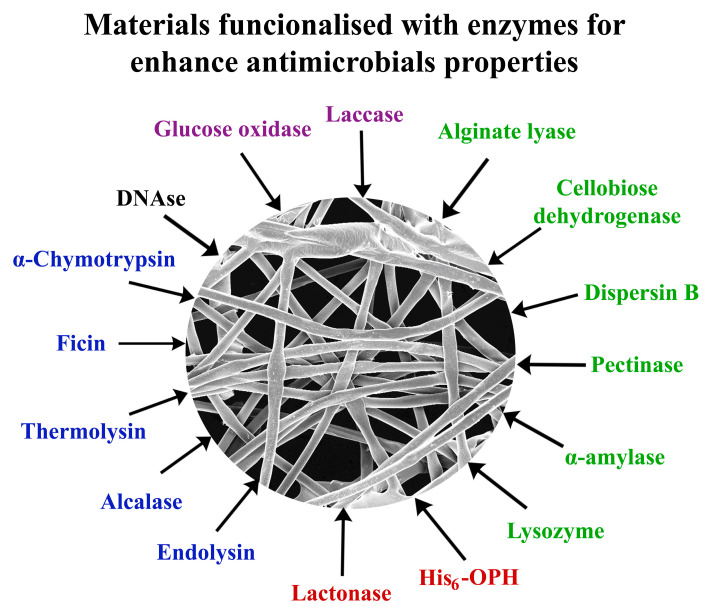
Enzyme used for the functionalization of various materials to enhance their antimicrobial properties.

The analysis of current trends in the functionalization of various materials due to the introduction of different enzymes in them (Table 1 [16,17,18,19,20,21,22,23,24,25,26,27,28,29,30,31,32,33,34,35,36,37,38,39,40,41], Figure 1) allows us to make several generalizations at the initial stage at once. First of all, this concerns the enzymes used. Their diversity is not too wide; mainly these are enzymes that belong to the class of oxidoreductases [16,17,18,19,20,28,35,36] and hydrolases [21,22,23,24,25,26,27,28,29,30,31,32,33,34,37,38,39,40,41]. Peptidases [21,31,34,38], various carbohydrases [22,23,24,25,26,27,29,30,32,33,34,39,40], and esterases [37,41] are actively used among hydrolases for obtaining materials with antimicrobial properties (Figure 1).

The main action of the used enzymes is aimed at inhibiting cell growth, assembling their structural elements, primarily the cell wall, as well as neutralizing the actions of the Quorum Sensing (QS)-signaling molecules of cells, which mainly transfer bacteria to the status of high resistance to the effects of antimicrobial agents [16,17,18,19,20,24,25,26,30,37,41]. 

In addition, the action of enzymes themselves or in the combinations with other biocides in the composition of functionalized materials contributes to the destruction of the formed biofilms due to the effective hydrolysis of exopolysaccharides that hold cells in dense cell populations and in a well-adhered state [21,22,23,30,31,32,33,34].

Among the obvious approaches implemented by different researchers to impart new or to improve already existing antimicrobial properties of various applied materials, the following should be highlighted. Firstly, concerns on the use of enzymes capable of generating products that have the potential to suppress the growth of microbial cells since they are effective oxidants (as in the case of H_2_O_2_) or substances that change the acidity of the media (as in the case of organic acids, in particular, gluconic acid) in the environment of treated cells. Secondly, the catalytic action of enzymes can be combined with the antimicrobial action of antibiotics [30,31,32,33,34], nanoparticles exhibiting an antimicrobial effect [37,39,40,41], with antimicrobial peptides [36,37,38] and with other enzymes [25,26,27,28,29]. Enzymes that exhibit multiple catalytic activities, such as ficin, which, being a sulfhydryl protease, demonstrates peroxidase-like activity [34], are of the greatest interest. 

It is interesting to note that among the targets for the effects of materials functionalized by enzymes, there are both Gram-positive [16,17,18,20,23,25,26,27,28,29,30,31,34,35,37,39,40,41] and Gram-negative [16,17,18,19,21,22,23,24,26,31,32,33,35,36,37,38,39,40,41] bacteria, as well as yeast cells mainly of the genus *Candida* [26,28]. 

Various especially successful variants of antimicrobial materials, which were developed with the introduction of enzymes in them, are those that have multiple activities in terms of suppressing the growth of various cells [16,17,18,23,26,27,28,31,35,37,39,40,41]. Such a wide range of objects of influence is achieved through the use, first of all, of broad-spectrum antibiotics, including the so-called “last reserve” antibiotics [30,31], the effectiveness of which just increases due to the manifestation of catalytic activities by enzymes.

**Table 1:** The antimicrobial materials functionalized with enzymes.

Enzymes	Material	Antimicrobial Properties	Effect of Enzyme Presence on Microorganisms
**Enzymes**			
Glucose oxidase (GOx) [16]	Cellulose beads	Growth inhibition of *Pseudomonas aeruginosa*, *Escherichia coli*, *Staphylococcus aureus*	Oxidative cell damage by H_2_O_2_
GOx [17]	Electrospun chitosan mats	Growth inhibition of *E. coli* and *S. aureus*	
GOx [18]	Polyester	Inhibition of *S*. *epidermidis* and *E. coli* growth	
GOx [19]	Poly(vinyl alcohol)/polycaprolactone multilayer system membrane	Inhibition of the *E. coli* growth	
GOx [20]	Chitosan with magnetic nanoparticle	Inhibition of proliferation of *S. aureus* suspended cells and biofilms	
α-Chymotrypsin [21]	Low-density polyethylene	Significant decrease in *E. coli* biofilm formation, reducing the number of adhered cells (up to 70.7%) and the matrix polysaccharide bio-volume (up to 80%)	Degradation of bacterial biofilms
Glycoside hydrolase [22]	Silica glass, polydimethylsiloxane and polystyrene	Significant reduce of surface attachment and *P. aeruginosa* biofilm formation (3-log reduction in surface associated bacteria)	Preventing of biofilm formation due to hydrolysis of poly-β-1,6-N-acetyl glucosamine
Glycoside hydrolase (Dispersin B) [23]	Fe_3_O_4_@SiO_2_	60% and 40% removal of *S. aureus* and *Bacillus cereus* biofilms; insignificant degradation of *P. putida* biofilms	Degradation of polysaccharides in the biofilms
Lysozyme [24]	Wool	Reduce the concentration of cells *E. coli* up to 95%	Destruction of bacterial cell wall
Lysozyme and tyrosinase [25]	Polyamide	Growth inhibition of *Micrococcus lysodeikticus*	
α-Amylase and alkaline pectinase [26]	Cotton	* MIC is 1156.3 μg/mL for *S. aureus*, 1156.3 μg/mL for *S. epidermidis*, 1156.3 μg/mL for *E. coli*, 18,500 μg/mL for *P. aeruginosa* and 4625 μg/mL for *Candida albicans*	
α-Amylase and lysozyme [27]	Polyethersulfone membrane	Decrease in formation of *S. aureus* and *S. epidermidis* biofilms	Prevention of attachment of microorganisms to the surface
Cellobiose dehydrogenase and deoxyribonuclease I [28]	Chitosan	Penetration through the matrix of polymicrobial biofilms of *C. albicans* and *S. aureus* and affect the embedded microbial cells	Disruption of the biofilm formation through degradation of extracellular DNA as a structural component of the formed biofilms
Dispersin B and endolysin SAL-1 [29]	Recombinant spider silk	Bacteriolytic effect and inhibition of *S. aureus* biofilm formation	Lysis of bacterial cells
**Enzymes with antibiotics**			
Lysozyme in combination with nisin [30]	Nanocrystalline cellulose	Growth inhibition of *Bacillus subtilis* and *S. aureus*	Destruction of bacterial cell wall; reduction in inhibitory concentration compared to lysozyme and nisin in free forms
Alcalase in combination with ciprofloxacin [31]	Carbopol Aqua SF1 nanogel	6-fold decrease in the biofilm mass and 3-log reduction in bacterial cells: *S. aureus*, *Pseudomonas aeruginosa*, *S. epidermidis*, *Klebsiella pneumoniae*, *E. coli*, *Enterococcus faecalis*	Degradation of bacterial biofilms and boosting of antibiotic action
Alginate lyase in combination with ciprofloxacin [32]	Chitosan	Significant reduction in *P. aeruginosa* biofilm aggregation; MIC is 0.125 μg/mL	
Alginate lyase in combination with ceftazidime or amikacin [33]		Inhibition of *P. aeruginosa* biofilm formation; MIC is 64 mg/mL	
Ficin with gentamicin, ciprofloxacin or benzalkonium chloride [34]		3-log reduction in *S. aureus* cell concentration	
**Enzymes with polyphenols**			
Laccase with poly(catechol) and poly- (p-phenylenediamine) [35]	Cotton, wool, and polyethylene terephthalate	10-100 fold decrease in both *E. coli* and *S. aureus* cell concentration	Catechol and *p*-phenylenediamine polymerization
**Enzymes with antimicrobial peptides**			
Laccase with KLWWMIRRWG- bromophenylalanine-3,4-dihydroxyphenylalanine-G and KLWWMIRRWG- bromophenylalanine-G [36]	Polystyrene	Inhibition of *E. coli* growth; MIC is 100 μg/mL	Increasing amounts of functional groups for immobilization of antimicrobial peptides
His_6_-OPH–polyelectrolyte complexes (polyglutamic acid (PLE_50_) with polymyxins [37]	Fibrous materials	Complex of polymyxin B with His_6_-OPH decreases the viability of both *B. subtilis* and *E. coli* cells	Hydrolysis of QS-signaling molecules and boosting of antibiotic action
Thermolysin in combination with polymyxin B [38]	Bacterial cellulose	Inhibition of *Pseudomonas* sp. growth	Thermolysin modified polymyxin confirmation and improved its antibacterial action
**Enzymes with metal nanoparticles**			
α-Amylase [39]	Ag–enzyme nanoaggregates	5.4 and 6.1 log reduction in *S. aureus* and *E. coli* cell concentration, respectively; 80% removal of cell biofilms	Degradation of the polysaccharides in biofilms and reducing cell attachment
α-Amylase [40]	Ag-nanoparticles	Significant inhibition of *Klebsiella pneumoniae* and *S. aureus* biofilm formation	Boosting of Ag-nanoparticles’ antibacterial action
His_6_-OPH–polyelectrolyte complexes (PEGylated derivatives of polyglutamic acid, PEG-PLE_50_) with Zn or Ta nanoparticles [37]	Fibrous materials (70% viscose and 30% polyester); activated carbon layer between polyester nonwoven fabrics (30% cotton and 70% meta polyaramide); fiber covered by poly(vinylidene difluoride)-co-poly(tetrafluoro- ethylene) membrane	Gradually decreasing of concentrations of viable *B. subtilis* and *E. coli* cells	Ta nanoparticles in combination with His_6_-OPH significantly increased the rate of cell death
His_6_-OPH/PLE_50_ [41] with Ta nanoparticles	Bacterial cellulose or fibrous materials (70% viscose and 30% polyester) modified by poly(4-hydroxybutyrate	Bacterial death (*B. subtilis*, *E. coli*), especially in the case of *E. coli* cells (up to 9-fold)	Synergetic effects of His_6_-OPH with Ta nanoparticles result in up to 4-fold harder elimination of bacterial cells

* Minimum inhibitory concentration (MIC).

Another general conclusion that was formed after the analysis of information about the introduction of enzymes in antimicrobial materials is related to the need for enzyme stabilization. The reason is that the components of the combinations being created can affect, among other things, the catalytic action of enzymes, and the protein molecules of enzymes themselves can affect the effectiveness of action of antimicrobial agents on cells [30,37,41]. In this regard, in a number of well-known solutions for the creation of enzyme-containing antimicrobial materials, a number of enzymes is immobilized covalently on the materials themselves [17,18,19,20] through the use of crosslinking agents and various chemical agents used to modify the surface of materials. This makes it possible to give greater stability to the action of enzymes and the functioning of antimicrobial materials themselves [17,18,19,20]. At the same time, such covalent crosslinking in the bulk of the material often leads to a change in the mechanical strength of the material itself; to hardening in water-containing media [17] or to an increase in brittleness and a decrease in elasticity in the air [20].Obviously, the area of application of different antimicrobial materials containing enzymes depends on the properties of the materials used; however, the efficiency of their action is completely determined by the characteristics of their enzymatic content. In this sense, the special properties of enzymes recently used for the development of functionalization of various materials will be analyzed.

### 2.1. Oxidoreductases in the Functionalized Materials with Antimicrobial Activity

As for the peculiarities of using different materials to obtain materials functionalized by different enzymes, it should be noted that almost every specific variant of the material used has its own nuances that are important for obtaining positive results. Thus, the oxidation degree and pH used for the enzyme-binding process were the most significant factors affecting the functionalization of cellulose particles by glucose oxidase (GOx). Slightly acidic environmental conditions (pH 6) during functionalization provided the best results for combination with GOx (Figure 1). An inhibitory effect of cellulose beads with glucose oxidase (GOx) was revealed for *Pseudomonas aeruginosa*, *Escherichia coli* cells and methicillin-resistant *Staphylococcus aureus* [16]. GOx is one of the most commonly used enzymes for the production of antibacterial materials without the addition of other biocides. 

Laccase (Figure 1) as an oxidoreductase may also be involved in the development of antibacterial materials, but it is used for the polymerization of polyphenols (catechol and *p*-phenylenediamine) for enhancing the material’s coating efficiency [35] or for the modification of polymeric surfaces for the attachment of bioactive compounds [36].

GOx (EC 1.1.3.4) is a flavoprotein that catalyzes the dehydrogenation of β-D-glucose to gluconic acid and H_2_O_2_ using molecular oxygen as an electron acceptor, and FAD acts as a redox transporter. GOx is attractive because it can be obtained from various microbial sources (*Penicillium* and *Aspergillus*) [42], including recombinant strains [43]. The antimicrobial activity of GOx is manifested in the ability to inhibit the growth of certain types of bacteria and fungi due to the products formed (H_2_O_2_ and gluconic acid, which lowers the pH of the medium) [18].

The initially obtained electrospun chitosan mats were functionalized due to the covalent immobilization of GOx. The obtained material with antimicrobial activity, owing to the action of GOx, steadily generated H_2_O_2_ (~60 µM/cm^2^), which led to the inhibition of the growth of both *E. coli* and *S. aureus* cells during 2 h of exposure of the material with cells [17].

Another variant of covalent immobilization of GOx was realized with the construction of a non-woven material with antimicrobial properties, when poly(ethylene terephthalate) was used as a functionalized base after its modification by plasma treatment or chemical inoculation of hyper-branched dendrimers [poly(ethylene glycol) or polyamidoamine] [18]. The results showed that the immobilized enzyme demonstrated improved thermal stability in the composition of such a material and retained 50% of its initial antibacterial activity after it was used six times against strains of pathogenic bacteria *Staphylococcus epidermidis* and *E. coli* in the presence of oxygen and glucose [18].

The immobilization of GOx in a poly(vinyl alcohol) membrane by encapsulating the enzyme inside the membrane, which, in turn, was protected by two hydrophobic polycaprolactone membranes, supplied the obtained materials with antimicrobial properties against *E. coli* cells, but at the same time that reduced the mechanical strength of membranes. Such an effect was associated with the increased polydispersity of the obtained fibers [19]. However, the addition of the GOx enzyme reduced the mechanical properties of poly(vinyl alcohol)-based membrane.

Gel matrices are often used to impart antimicrobial properties to them, including by immobilizing enzymes. Thus, the biocompatible chitosan nanoparticles obtained by ionotropic gelation were used for the covalent immobilization of GOx. The immobilized enzyme obtained in this way improved its stability, and the resulting nanoparticles were well dispersed in different media, providing the effective inhibition of the reproduction of *S. aureus* cells taken for study in suspension or as a part of formed biofilms [20].

**Figure 2:**
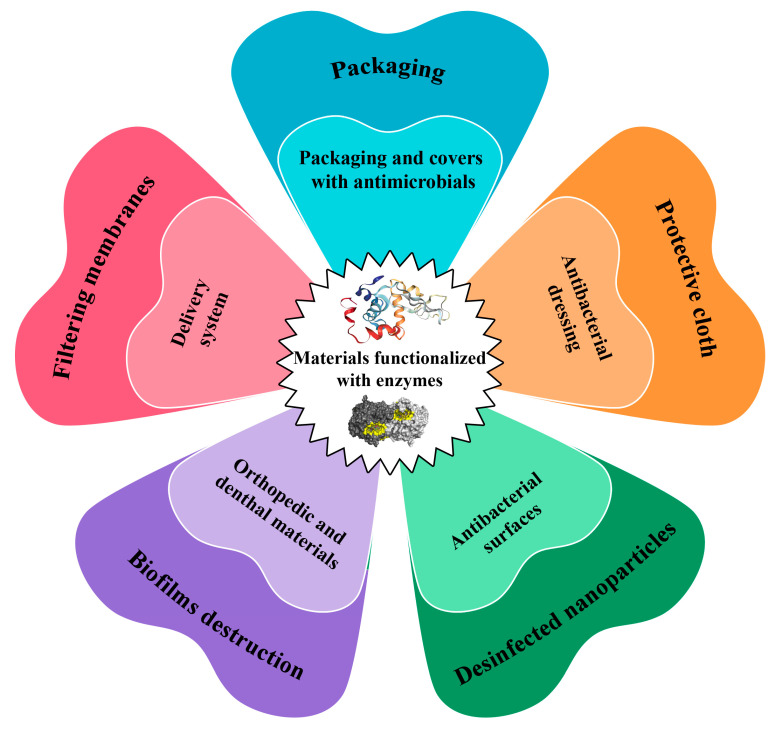
Main applications of the antimicrobial materials functionalized by different enzymes.

Today, various wound dressings based on hydrogels containing GOx are developed for the treatment of skin infections (Figure 2). For example, GOx@F68/F127F68/F127 hydrogel was obtained based on biocompatible Pluronic F-68 and Pluronic F-127 with glucose oxidase. The obtained material inhibited the growth of both Gram-positive and Gram-positive bacterial strains in vitro and in vivo. The degree of antibacterial action of GOx@F68/F127 reached 100% against both *S. aureus* and *E. coli* [44].

Bacterial/fungal biofilm persistent infections are some of the most frequent clinical lesions of the oral cavity, leading to apical periodontitis and tooth damage. Conventional disinfectants used to treat root canals are harmful to the teeth and tissues, which makes them ineffective in the treatment of these lesions. The development of new antibacterials is an important scientific and practical task. Magnetic nanoparticles (MNP) modified by GOx were obtained. The GOx-MNP, which had excellent biocompatibility and compatibility with blood, were characterized by the high efficiency of antibacterial action against *Enterococcus faecalis* and *Candida albicans* cells. It is important to note that upon contact with bacterial/yeast biofilms, the dense matrix of the biofilm was destroyed due to the movement of GOx-MNP induced by the magnetic field and the formation of reactive oxygen species generated by GOx [45].

Thus, the antibacterial properties of materials with GOx are manifested in the production of H_2_O_2_ and the accumulation of gluconic acid. Hydrogen peroxide kills bacterial cells due to the peroxidation and destruction of cell membranes, and gluconic acid reduces the pH of the medium, which also negatively affects the growth of bacteria. As a result, a complex antimicrobial effect is observed.

### 2.2. Carbohydrases in the Functionalized Materials with Antimicrobial Activity

The destruction of bacterial biofilms is still a huge problem. Current strategies are based on obtaining antibacterial and anti-adhesive coatings functionalized by enzymes hydrolyzing peptidoglycan in bacterial cell walls. Among such enzymes, lysozyme (EC 3.2.1.17) (Figure 1), an enzyme of the hydrolase class that accelerates the hydrolysis of the β(1→4)-glycoside bond between *N*-acetylmuramic acid and *N*-acetylglucosamine in the peptidoglycan of the bacterial cell wall, has become more widespread [24,25]. The reason for the popularity of lysozyme for material functionalization is its thermal stability over a wide temperature range and antimicrobial activity against a wide range of bacteria and fungi; however, it naturally exhibits maximum antimicrobial activity against Gram-positive bacteria [46].

#### 2.2.1. Lysozyme

Recently, the antimicrobial properties of lysozyme immobilized on bacterial cellulose nanofibers were investigated [46]. Such antimicrobial material turned out to have good characteristics, since the activity of the enzyme decreased by only 12% and 30% during immobilization and after prolonged periodic use (in nine working cycles). At the same time, the antimicrobial activity of lysozyme against a number of cells (*Staphylococcus aureus*, *Escherichia coli*, *Listeria monocytogenes*, *Yersinia entercolitica*, *Aspergillus niger* and *Saccharomyces cerevisiae*) increased after immobilization. The maximum antimicrobial effect was observed for lysozyme, introduced in this way into the functional material, on *A. niger* and *S. cerevisiae* cells [46].

The covalent immobilization of an enzyme with antimicrobial activity during the functionalization of different materials is used for lysozyme as actively as for GOx [24,25]. Tris(hydroxymethyl)phosphine was used as a crosslinking agent to immobilize lysozyme on the surface of wool fiber [24]. Biocide activity was shown against *E. coli* cells with a 95% reduction in the number of bacterial cells, and the size of the inhibition zone was 4.5 mm, which indicated that a good antibacterial effect was obtained. Wool samples with an immobilized enzyme showed a gradually decreasing (up to 73%) antibacterial activity after five washing cycles.

An interesting result was obtained when creating an antibacterial material based on lysozyme in combination with nisin, which were immobilized on nanocrystalline cellulose functionalized by aldehyde groups [30]. The material retained the antimicrobial activity acquired due to lysozyme for quite a long time, but it turned out that the minimum inhibitory concentrations (MIC) of conjugated nanocellulose were higher in relation to *Bacillus subtilis* (200 ppm) and *Staphylococcus aureus* cells (50 ppm) than in lysozyme and nisin in their soluble form (50 and 32 ppm, respectively). The possible reason was that immobilization affected the spatial orientation of these proteins, limiting the active center for binding to polymer substrates.

It should be noted here that, in general, the combination of lysozyme with nisin to prevent food spoilage has been used by other researchers who have shown that the effect of the combination of lysozyme and nisin on *S. aureus* cells is more effective at pH 5.5 (MIC is 19 µg/mL) [47]. The effect of the combination of the antibiotic with the enzyme on *E. coli* cells was more pronounced than the effect of each of them separately, especially at pH 5.7 and 8.0. However, in the case of *Listeria monocytogenes*, this effect was much weaker. The effect against *Salmonella typhimurium* was better at pH 5.5 and 6.0.

Thus, it is necessary not only to select the material and method for the immobilization of lysozyme, but also to select the conditions for its use to affect various bacterial contaminations.

Recently, a new material was developed based on nanofibrillated cellulose with lysozyme to accelerate wound healing. The patches obtained from such a material had antioxidant and antimicrobial activity against *S. aureus* (a decrease of 3.5 log CFU/mL was observed). Such materials proved to be well biocompatible with L929 fibroblast cells, and in vitro wound healing analysis showed good migration ability, leading to almost complete occlusion of wounds [48].

Using the molecular imprinting method, supermacroporous cryogel membranes with lysozyme have recently been manufactured, which can potentially be used for various purposes: for wound dressing or for food packaging [49]. The methyl ester of *N*-methacryloyl-(L)-histidine (MAH) was used as a pseudospecific ligand and formed a complex with Cu^2+^ to ensure the coordination of metal ions between MAH and the lysozyme molecule. Poly(hydroxyethylmethacrylate-*N*-methacryloyl-(L)-histidine) P(HEMA-MAH) was used for the synthesis of the cryogel membrane, the free radical polymerization of which was initiated by N,N,N′,N′-tetramethylenediamine (TEMED) and ammonium persulfate. Further, an unexpected result was obtained for this material since it was found that the antimicrobial action of cryogel membranes was more pronounced against Gram-negative *E. coli* bacteria as compared to Gram-positive cells because 60% lysis of *E. coli* cells was noted on the third hour of contact with cells, whereas for *S. aureus* the decrease in the number of cells was two times less. The same difference of two times less was preserved on the sixth hour of incubation of cells with a material containing lysozyme when 100% death of *E. coli* was recorded. In this case, the MAH-Cu complex acted as a metal chelator and enhanced the antibacterial effect of lysozyme against Gram-negative bacteria by destabilizing their outer membrane.

The development of new technologies for applying paints to fabrics makes it possible to use paints with different compositions, in particular, to introduce different enzymes in them and to immobilize enzymes on materials using inkjet printers. For example, samples of printed fabrics were obtained from plasma-treated polyamide-6,6 with lysozyme loaded to it [25]. At the same time, the enzyme tyrosinase was applied alternately with lysozyme to the same tissue, which served as a “seamstress” in relation to lysozyme, since tyrosinase “sewed” it to polyamide through tyrosine residues on the lysozyme protein molecule. The lysozyme, thus, bound to the printed fabrics showed quite satisfactory antimicrobial activity when the *Micrococcus lysodeikticus* cells were used as a bacterial object for its growth suppression, and the tissue retained about 24% of the initial enzyme activity for up to four repeated uses. The tissue retained almost 50% of its original antimicrobial activity when stored in the cold for 1 month.

Thus, lysozyme, due to its high antibacterial activity against many pathogenic bacteria, is currently one of the most used enzymes for the functionalization of biomaterials. 

#### 2.2.2. α-Amylase and Pectinase

Biofilms, in which bacteria acquire high resistance to the effects of antimicrobial agents, are formed with the participation of exopolysaccharides synthesized by highly concentrated cell populations [50]. One of the most effective ways to eliminate biofilms is their destruction by using enzymes capable of hydrolysis of polysaccharide components and weakening of the biofilm matrix. Such enzymes include glycoside hydrolases. Among these enzymes, in recent studies, special attention has been paid to the use of α-Amylase, Dispersin B and alginate lyase to functionalize various materials and give them antimicrobial properties (Table 1, Figure 1).

The functionalization of materials consists of the immobilization of enzymes on their surfaces and the formation of chemical (hydrogen, hydrophobic, electrostatic, and ionic) interactions between the enzymes and carriers, enabling the maintenance of high catalytic activity of the enzymes. The most common method of the immobilization of enzymes for the functionalization of materials is adsorption. Due to its simplicity and low cost, this method is successfully applied in practice [51]. Covalent immobilization is also used, and it involves the formation of strong and stable chemical bonds between the enzyme and the carrier. That limits the leaching of the enzyme and prolongs its active functioning [51]. The stability of immobilized enzymes makes it possible to destroy the polysaccharides in biofilms or in the cell walls of bacteria, preventing the anchoring of microorganisms to the surface [27] and preventing the action of such biocidal agents as silver nanoparticles [39,40].

α-Amylase (EC 3.2.1.1) hydrolyzes α-1,4-glycoside bonds, and therefore, it is interesting for modifying the properties of functional materials [26,27]. The enzyme combines well with nanomaterials exhibiting biocidal activity (CuO, ZnO, Ag, and Cu), which allows obtaining products with a synergistic effect. Thus, a catalytic system containing α-amylase immobilized on ZnO nanoparticles was created [51]. The resulting combination showed antibacterial activity against both Gram-positive and Gram-negative bacteria. α-Amylase degrades polysaccharides in the bacterial cell wall, and ZnO nanoparticles are involved in generating oxidative stress due to the formation of reactive oxygen species. In addition, the nanoparticles can agglomerate on the surface of the bacteria, affecting trans-membrane processes.

The fungicidal properties of the nanoparticles were limited, and the addition of α-amylase improved the biological activity of the resulting combination. The immobilized enzyme also showed higher activity than in the free form [51]. It should be noted that α-amylase from *Bacillus subtilis* is known for its ability to inhibit the development of biofilms formed by pathogens *P. aeruginosa* and *S. aureus*. At the same time, the destruction of these biofilms requires maintaining pH 8.0 for 6 h [52].

It should be noted that several developments are known for α-amylase in which researchers combined this enzyme with other enzymes to enhance the antibiotic activity of the obtained materials. So the combination of α-amylase from *Bacillus* sp. with protease from the pancreas of cattle type I (PtI) it is known to prevent the formation of bacterial biofilms. The combination of enzymes made it possible to achieve the inhibition of biofilm development by 78% and 90% against *E. coli* and *S. aureu*, respectively [53].

Antibacterial membrane materials reducing biofilm formation have been developed. α-Amylase and lysozyme, as antibacterial enzymes, were covalently immobilized on polyethersulfone membranes functionalized with polydophamine/cyanuric chloride. That resulted in the formation of biocompatible antibacterial surfaces [27]. Such a multilevel protective system prevented the contamination of the membrane and the formation of biofilms. The results of the microtiter test and flow cytometry showed that the samples of such membranes were guaranteed to remove biofilms by more than 87%

It is known that pectinases isolated from *Pseudomonas stutzeri* are able to inhibit biofilms and reduce the adhesive properties of different cells: in *Pseudomonas aeruginosa* by 72% and 37%, respectively, in *Enterococcus faecalis* by 67% and 30%, and in *Staphylococcus aureus* by 53% and 28%, respectively [54]. Therefore, there is an interest in pectinases as enzymes with which an antimicrobial combination can be created for the functionalization of different materials. 

An interesting study was undertaken in which thermally stable α-amylase (Termamyl ® 2X) and alkaline pectinase (Bioprep ® 3000L) were compared in their antimicrobial activity [26]. It appeared that alkaline pectinase proved to be more active compared to Thermamil ® 2X against Staphylococcus aureus, epidermal *Staphylococcus*, *E. coli* and *Candida albicans* cells, and it was also partially active against *Pseudomonas aeruginosa*, demonstrating a high potential for use in the production of antimicrobial fabrics. In addition, the enzyme Bioprep ® 3000L covalently immobilized on the surface of chemically modified cotton fabric proved to be more effective against all mentioned microorganisms.

Numerous combinations of antibacterial agents with enzymes are effective solutions used today to create new materials with antimicrobial properties. A hydrogel consisting of aminoglycoside antibiotics, pectinase and oxidized dextran was developed for the treatment of infections associated with antibacterial biofilms [55]. The primary amines of aminoglycosides and pectinases reacted with aldehyde groups on oxidized dextran via a pH-sensitive Schiff bond to form a hydrogel. In bacterial infection, increased acidity causes the release of both pectinase and aminoglycoside antibiotics. The released pectinase effectively cleaves extracellular polysaccharides surrounding bacteria in the biofilm, and thus, increases the sensitivity of bacteria to aminoglycosides.

Thereby, to obtain antimicrobial materials, amylase and pectinase are used in combination with biocidal agents to remove biofilms and prevent the adhesion of bacterial cells to treated surfaces.

#### 2.2.3. Dispersin B and Alginate Lyase

Among glycoside hydrolases, there are several other enzymes [22,23,29] that are involved in the production of antimicrobial materials (Figure 1), in particular, dispersin B (DspB), which is produced by *Aggregatibacter actinomycetemcomitans* cells. This enzyme was used to produce a multicomponent coating with an antibacterial and antibiotic film surface. Thin films containing dispersin B, which destroys exopolysaccharides necessary for cells to form biofilms and silver nanoparticles with an antimicrobial effect, were obtained. At the same time, it turned out that similar coatings containing only silver do not interfere with the adhesion of bacteria and the formation of biofilms, which confirmed the feasibility of combining silver with this glycoside hydrolase to obtain surfaces with double action [56]. Immobilization of the same dispersin B on magnetic nanoparticles Fe_3_O_4_@SiO_2_ as a magnetoreceptor (MagR) [23] gave the possibility of the composite material purification from biofilms not only due to the presence of glycoside hydrolase, but also due to the possible application of a magnetic field on such materials (DspB-MagR). This approach forms a possibility of creating self-cleaning surfaces with medical devices (Figure 2).

Materials functionalized by different hydrolytic enzymes (dispersin B and two endolysins PlySs2 and SAL-1) were effective in their action in the frame of materials functionalized by them [54,55,56,57]. The enzymes that were introduced in the coatings formed from recombinant spider silk protein [29], enabled obtaining an effective composite material with a bacteriolytic effect on *Staphylococcus aureus* cells. Such functional coatings can be used to reduce the risks of osteoblast formation during the installation of orthopedic and dental implants (Figure 2).

Alginate is a key component of exopolysaccharides in the bacterial biofilm of *P. aeruginosa*, which is formed in patients with cystic fibrosis [32,33]; therefore, one of the strategies to combat such formations is the use of alginate lyase [32,33]. According to the classical nomenclature based on the specificity of action, endoalginate lyases are divided into polyguluronate lyases (EC 4.2.2.11), polymannuronate lyases (CF 4.2.2.3) and bifunctional alginate lyases specific to 1→4-glycoside bonds between the residues of both mannuronic and guluronic acids. There are contradictory reports in the literature about the effectiveness of the use of these enzymes against biofilms and their synergistic action with antibiotics [32,33]. However, it was found that only enzymes with polyM/G activity, such as Alg2A and A1-II′ (alginate lyase from *Sphingomonas* sp.), are effective in the destruction of biofilms [58]. They were immobilized on chitosan nanoparticles with ciprofloxacin for the effective treatment of infection accompanied by the formation of the *P. aeruginosa* biofilm [32], and the presence of the enzymes improved the effect of the antibiotic.

In general, enzymes that destroy polysaccharides used in combination with antibiotics, metal nanoparticles and antimicrobial peptides have made it possible to achieve noticeable positive results in the fight against bacterial biofilms.

### 2.3. Proteolytic Enzymes in the Functionalized Materials with Antimicrobial Activity

In bacterial biofilms, cells, in addition to polysaccharides, are surrounded by extracellular proteins, nucleic acids and lipids, which are secreted by cells, and chemically differed in various types of bacterial cells. To reduce the risks of infections associated with the formation of biofilms, proteolytic enzymes (Figure 1) are also used [21,29,31,34]. These enzymes (EC 3.4) belong to the class of hydrolases and cleave peptide bonds between amino acids in proteins and peptides and are divided into exopeptidases and endopeptidases. Proteolytic enzymes, initially obtained from plant, animal and microbial sources, are widely used, including in medicine, food and the feed industry [59,60]. The antibiotic film potential of proteases was studied using the example of α-chymotrypsin and ficin [21,34]. It was found that in addition to the hydrolysis of extracellular proteins involved in the formation of biofilms, proteases can disrupt intercellular communication due to the lysis of signal peptidase type I [31]. 

#### 2.3.1. α-Chymotrypsin and Ficin

Proteases and peptidases are also actively used to functionalize various materials and give them antimicrobial properties alone [21] or in combination with other enzymes [29] and antibiotics [31,34]. The use of covalently immobilized α-chymotrypsin (EC 3.4.21.1) for these purposes is considered traditional since such materials have been created for many years, and not only recently. It is possible to note a change only in the materials that are themselves subject to functionalization with the use of chymotrypsin, in particular, the use of this enzyme for introduction into packaging materials, for example, in low-density polyethylene (LDPE) [21]. 

Ficin (EC 3.4.22.3) (Figure 1), plant enzyme, is another interesting object for the use of hydrolytic enzymes in the development of antibacterial materials. It is a sulfhydryl protease isolated from fig tree latex, which can hydrolyze peptide bonds formed with the participation of a large number of amino acids (methionine, lysine, arginine, glycine, serine, threonine, valine, asparagine, alanine and tyrosine). It has been proven that ficin is a bifunctional enzyme that has peptidase and peroxidase-like activity in the destruction of *S. aureus*, *S. biofilms*, *S.epidermidis*, *S. mutans* [61]. It transpired that this enzyme in the composition of functionalized materials perfectly combines with various antibiotics and antimicrobial agents, including gentamicin, ciprofloxacin, benzalkonium chloride [34], and dalbavancin [62]. The use of chitosan materials for the manufacture of wound dressings with ficin and antibiotics allows, in vivo, the acceleration of the healing of wounds infected with *S. aureus* twice as fast in comparison with the absence of ficin. These are very promising results for practical ficin applications (Figure 2).

#### 2.3.2. Endolysins

Bacteriophages are now actively used to produce enzymes that are considered alternatives to antibiotics or partners for a successful combination with them. Some endolysins exhibit multiple catalytic (muramidase, endopeptidase, and amidase) activity aimed at cell destruction [63]. The popularity of endolysins among proteolytic enzymes used for obtaining materials with antimicrobial action is clearly growing [29,64,65,66]. The reason is that endolysins (Figure 1) are phage-encoded hydrolytic enzymes released at the final stages of the replication cycle to hydrolyze the peptide bond between D-glutamine and L-alanine in the peptidoglycan of the bacterial cell wall. Thus, the antibacterial activity of LysE endolysin against *Aeromonas hydrophila* cells [64], LysMR-5 endolysin against cells of polyresistant strains of *S. aureus* and *S. epidermidis* [65], and LysP108 against *S. aureus* and *P. aeruginosa* cells [66] was established. The range of materials used today for their functionalization by endolysins is expanding, especially due to the combination of these enzymes with antibiotics and other enzymes while varying materials based on natural polymers [29,65]. Obtaining recombinant endolysins reduces their cost and the materials containing them, making them more attractive for a wide application.

Thus, the use of proteolytic enzymes in combination with known antibiotics and other enzymes makes it possible to successfully suppress the formation and growth of bacterial biofilms. 

### 2.4. Quorum Quenching Enzymes in the Functionalized Materials with Antimicrobial Activity

One of the reasons for the antibiotic resistance of microbial cells is their ability to change their biochemical status as part of highly concentrated populations under the action of low-molecular-weight QS autoinducers. The QS autoinductor molecules in most Gram-negative and Gram-positive pathogenic bacteria are various molecules of *N*-acyl homoserine lactones (AHL) and molecules of oligopeptides, respectively [67]. In this regard, there is a huge interest in enzymes capable of destroying QS under the action of enzymes capable of catalyzing the hydrolysis of these molecules, leading to a significant weakening of the protective properties of cells (Figure 1).

Among the enzymes capable of catalyzing the hydrolysis of signaling oligopeptides, thermolysin (EC 3.4.24.27) attracts attention, which, being a thermostable proteinase, hydrolyzes peptide bonds formed by the residues of hydrophobic amino acids (isoleucine, leucine, valine, phenylalanine, methionine and alanine) and can be used to create antimicrobial materials against Gram-positive bacterial cells [38]. However, this enzyme has weak antimicrobial activity, so it is advisable to combine it with an antibiotic; for example, with polymyxin B. Loaded in this combination to bacterial cellulose, which has the potential to be used as a dressing material with a good absorbency, thermolysin noticeably improves the effect of the antibiotic action. Lactonases (EC 3.1.1) produced by various microorganisms have potential antibacterial properties mostly against Gram-negative pathogenic bacteria and catalyze the hydrolysis of AHL and fungal mycotoxins [68,69,70,71,72]. However, their use is most often limited by the thermal instability or low activity of these enzymes despite the presence of some thermostable variants; for example, natural lactonase (AidB) from *Bosea* sp. with an optimum at 60 °C [73] or mutant enzyme PPH-G55V, showing 50% activity at 62 °C [68]. In this regard, most of the known lactonases require a mandatory solution to the problem of their stabilization when they are introduced in different functionalized materials. 

Today, various approaches and solutions are known in this regard. For example, the encapsulation of lactonase in capsules consisting of the peptide tretbutoxycarbonyl-Phe-Phe-OH [68] or its introduction in the structure of the so-called nanoflowers (AhlX@Ni_3_(PO_4_)_2_ [72] is effectively used to combat *Erwinia amylovora* and *E. carotovora*, respectively; its introduction into the gel of carboxymethyl cellulose is known for its use against *P. aeruginosa* cells [69]; its complexation together with silver nanoparticles (AgNP) is known to suppress the development of biofilms with *Klebsiella pneumoniae* cells [70]. 

In the case of nanoflowers, the authors managed to achieve the best results in lactonase stabilization (although thermally stable lactonase was used in the experiments). As a result, the enzyme retained 100% activity when used for 40 days and 80% activity at 60 °C for 48 h. As it transpired, such nanoflowers with lactonase activity can be used for the treatment of various plants (potatoes, radishes, peppers, Peking cabbage and eggplants) to prevent their damage by Gram-negative soil bacterial cells *Burkholderia glumae* [72]. This result is an important sample of environmentally friendly problem solution.

Hexahistidine-containing organophosphorus hydrolase (His_6_-OPH) (EC 3.1.8.1) is one of the enzymes that are active against individual AHL molecules, catalyzing the rupture of the lactone ring [37,41,74]. It transpired that β-lactam antibiotics (ampicillin, meropenem, imipenem, ceftriaxone) [75,76] and various antimicrobial peptides (AMP) [77,78] (Table 2) can have a stabilizing effect on this enzyme due to the formation of non-covalent polyelectrolyte complexes with the enzyme molecule. 

It should be noted here that today AMP are considered potential alternatives to known antibiotics, which is why they are so popular in many recent studies. During the preparation of such AMP complexes with His_6_-OPH it transpired that they differ greatly in their lactonase activity and, accordingly, in their antimicrobial action (Table 2). Among the studied AMP and antibiotics of poly(amino acid) nature, Indolicidin became the champion in combination with His_6_-OPH for Gram-negative cells, and polymyxin B turned out to be the leader in the suppression of Gram-positive cells. 

A noticeable improvement (up to 2.8 times) was revealed in the antibacterial efficacy of bacitracin against Gram-negative cells when it was combined with His_6_-OPH (Table 2). It has been shown that the combination of His_6_-OPH/bacitracin against a number of yeast strains can significantly reduce (up to 9.3 times) the inhibitory concentration of the latter [78].

**Table 2:** Comparison of the inhibitory concentration (*IC*_10_^*^, mg/L) of different AMP with or without (w/o) His_6_-OPH combination against Gram-positive and Gram-negative bacteria [38,77,78].

AMP	With or w/o Bacterial Cellulose	w/o His_6_-OPH	With His_6_-OPH	Decrease (Times)
*Pseudomonas* sp.				
Bacitacin	–	5.27 ± 0.31	1.89 ± 0.06	2.79
Indolicidin	–	37.6 ± 1.9	0.24 ± 0.03	156.67
Temporin A	–	9.4 ± 0.7	0.41 ± 0.05	22.93
Colistin	+	0.92 ± 0.05	0.03 ± 0.004	30.67
Polymyxin B	+	0.87 ± 0.01	0.23 ± 0.03	3.78
Indolicidin	+	1.39 ± 0.03	0.52 ± 0.05	2.67
Temporin	+	0.45 ± 0.01	0.24 ± 0.004	1.87
*Bacillus subtilis*				
Bacitacin	–	0.02 ± 0.001	0.02 ± 0.002	1
Indolicidin	–	4.66 ± 0.61	2.62 ± 0.22	1.78
Temporin A	–	2.15 ± 0.31	0.60 ± 0.13	3.58
Colistin	+	1.23 ± 0.04	0.28 ± 0.006	4.39
Polymyxin B	+	1.03 ± 0.03	0.08 ± 0.002	12.87
Indolicidin	+	13.3 ± 0.81	2.45 ± 0.05	5.43
Temporin A	+	1.58 ± 0.01	1.06 ± 0.002	1.49

**Figure 3:**
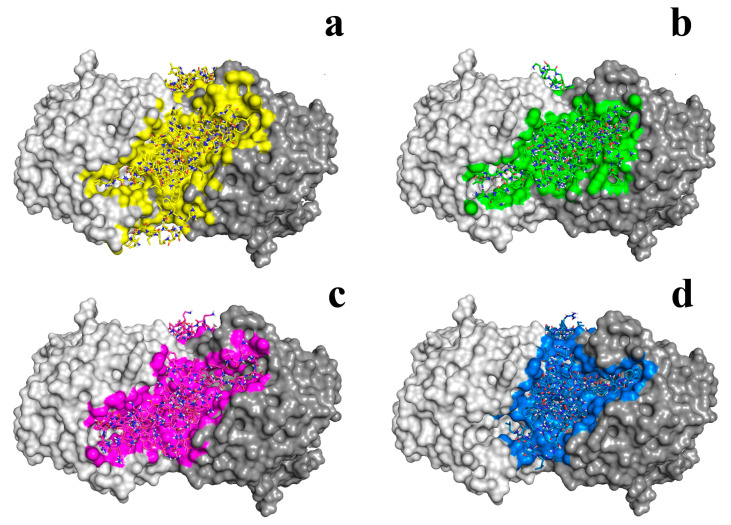
Active nanocomplexes of His_6_-OPH dimer with different AMP obtained in silico by molecular docking: (**a**) Bacitracin (yellow), (**b**) Colistin (green), (**c**) Polymixin B (violet), (**d**) Temporin A (blue). The “face” surface of the dimer molecule of the enzyme covered by molecules of AMP is marked in each case by special color.

Analysis of the causes of the incident using computer modeling methods (Figure 3) allowed us to determine the reasons for the differences between the properties of the obtained combinations of the enzyme with “partners” in polyelectrolyte complexes. Those AMP showed a negative effect on the activity of the enzyme, as it transpired, were localized on the surface of the His_6_-OPH dimer in such a way that they shielded the entrances to the enzymatic active centers of substrates (QS molecules) [76,77]. That was a reason that the combinations did not show any improvements in antimicrobial activity at all compared to a single action of AMP (without the enzyme). 

Similarly stabilized forms of His_6_-OPH obtained as part of complexes with antibiotics and poly(amino acids) were successfully used further for non-covalent modifications of various materials, giving them antimicrobial characteristics. So there were functionalizations of composite materials based on nanocellulose and poly(vinyl alcohol) cryogel [79] and various fibrous materials, including those of general consumption, containing 70% viscose and 30% polyester [37]. Interestingly, the use of stabilized forms of His_6_-OPH in the same materials, but with the stabilization of the enzyme not by polypeptide antibiotics, but by poly(amino acids) without antimicrobial properties, with the addition of metal nanoparticles (Zn or Ta) allowed the acquisition of even more effective variants of functionalized materials against *B. subtilis* and *E. coli* cells [37,41].

Nevertheless, it is necessary to emphasize that the helpful use of experiments in silico considerably simplified the choice of effective stabilizers for the enzyme used for the functionalization of materials and guaranteed the obtaining of improved antimicrobial effect. Thus, the possibility of using enzymes that suppress the QS of bacterial cells to produce anti-bacterial materials is currently being actively investigated. Additionally, the combination of such enzymes with antimicrobial polypeptides is considered an effective approach to improving the properties of known antibiotics or forming an alternative to them.

## 3. Metal–Organic Frameworks Functionalized by Enzymes as Antimicrobials

Metal–Organic Frameworks (MOF), which are coordination polymers in which metal ions are bound to each other using polydentate ligand linkers acting as electron donors for the metal, are becoming increasingly common for the production of materials with antibacterial properties. Residues of carboxylic acids (carboxylates) or nitrogen-containing heterocyclic compounds, often imidazolate ions, act as linkers. 

MOF with different characteristics can be obtained by adjusting the combination of metal nodes and organic ligands with different pore sizes, a variable structure and a large surface area. A huge number of MOF have already been synthesized and have found their application as sensors, drug delivery systems, catalysts, fuel cells, etc. [80,81]. MOF can be used to encapsulate antibacterial preparations, to release metal ions with antibacterial properties, and to immobilize enzymes. In addition, the organic ligands themselves used for MOF synthesis may have antibacterial activity [82]. 

The immobilization of enzymes in MOF can be carried out by encapsulation and infiltration and may surface bound through covalent bonds or non-covalent interactions. In order to increase antibacterial properties, MOF with peroxidase-like activity are mainly used today, effectively converting H_2_O_2_ into hydroxyl radicals with high toxicity to cells by immobilizing GOx [83]. Actually, there are a lot of exemplars of GOx actively functioning with MOF (Table 3, [83,84,85,86,87,88,89,90,91,92,93,94]).

MOF with GOx were developed as self-activated cascade hybrid nanoreactors (MIL@GOx-MIL NR). This provided the formation of gluconic acid and a decrease in pH to 4 at which MIL@GOx-MIL NR showed the highest activity of the cascade reaction. The continuous production of H_2_O_2_ was accompanied by the formation of highly toxic hydroxy-strong radicals (HO^−^) significantly inhibiting the growth and formation of *S. aureus* biofilm [83].

In another study, MOF from mesoporous cerium with GOx showed antibacterial properties against *E. coli* and *S. aureus* cells. In vivo experiments have demonstrated that obtained MOF can effectively destroy 99.9% of bacteria in wound tissues and prevent persistent inflammation without damaging normal tissues [84].

MOF (GOx@Fe-ZIF-TA) obtained on the basis of the zeolite imidazolate framework (ZIF) doped with Fe, encapsulated GOx and etched with tannic acid were used for the treatment of infected diabetic wounds. The obtained MOF showed satisfactory anti-bacterial activity against *E. coli* cells [85]. In the process of using MOF with GOx, gluconic acid and H_2_O_2_ were produced as usual. The MOF sample itself, containing Fe(II), also took part in oxidative catalysis. Thus, a chemical-biocatalytic oxidative “blow” was applied to the cells in this case of MOF.

MOF (MnFe_2_O_4_@MIL/Au&GOx) showed antibacterial activity against *E. coli* and *S. aureus* cells, continuously transforming glucose into gluconic acid and H_2_O_2_. Au nanoparticles were used to increase the catalytic peroxidase activity of MnFe_2_O_4_@MIL with the formation of a large number of reactive oxygen species (ROS). It has been shown that bacteria secreted glutathione (GSH) when ROS appeared, and MnFe_2_O_4_ nanoparticles could conduct GSH depletion, thereby destroying the bacterial intercellular defense system and further enhancing the antibacterial effect of ROS [86].

MOF (L-Arg/GOx@Cb DC) with the co-encapsulation of Gox and L-arginine (L-Arg) in CuBDC (Cu-terephthalate) with a catalytic activity similar to Fenton were used to achieve a synergistic antibacterial effect. Copper is the second most commonly used metal after iron in the heterogeneous Fenton process due to its redox behavior. Cu ions can react with H_2_O_2_ to form highly toxic hydroxyl. After glucose oxidation, a large number of toxic radicals ONOO^−^ and NO^−^ are synthesized by a two-stage reaction. As a result, 97% growth inhibition of *E. coli* and *S. aureus* cells can be achieved at 38 μg/mL MOF and lower concentration [92]. MOF, consisting of mesoporous nanoparticles Fe_3_O_4_@SiO_2_, GOx and L-arginine, had similar characteristics [93].

L-arginine was injected into MOF to form NO under the action of H_2_O_2_. The effectiveness of action of the MOF was demonstrated against *S. aureus* and *E. coli* bacteria when the used MOF concentration was 80 µg/mL. In addition, using an external magnetic field, this type of MOF could be evenly distributed over the entire depth of the infectious biofilm for it to be removed.

The samples of hybrid ultrathin two-dimensional (2D) MOF (2D Cu-TCPP(Fe)) with adsorbed GOx were developed for effective bacterial therapy in vivo. Glucose was continuously converted into gluconic acid and H_2_O_2_ under the action of GOx with a decrease in pH from 7 to 3-4, sharply activating the peroxidase activity of CR-TCPP(Fe) with the generation of extremely toxic OH^−^ resulting in the death of *E. coli* and *S. aureus* cells. In vitro and in vivo results showed that the developed self-activating cascade reagent had a reliable antibacterial effect with negligible biotoxicity [87].

Today, ROS are used in the treatment of bacterial infectious diseases in antibacterial chemodynamic therapy. However, the duration of ROS is too short to achieve complete cell death; therefore, residual concentrations of bacterial cells inevitably continue to proliferate and may become a source of superinfection. To solve this problem, an innovative bimetallic organometallic framework (BMOF) containing copper, zinc and GOx was developed by an electrostatic self-assembly method [88]. The GOx catalyzed the conversion of glucose into H_2_O_2_, and then the ions of Cu^2+^ converted it into OH^−^, which led to a highly effective bactericidal action of the MOF against *E. coli* and *S. aureus*. The study of such a MOF sample in vitro and in vivo confirmed its cytocompatibility and reliable ability to accelerate a post-infectious full-layer regeneration of the skin, contributing to epithelialization of the wound and angiogenesis.

Bacterial growth was also effectively suppressed by the MOF, obtained on the basis of a homologous zeolite imidazolate framework and immobilized GOx [89,90]. MOF (ZIF-ICG@ZIF-GOx@MPN) samples with GOx and indocyanine green (IGG) included in a zeolite imidazolate framework (ZIF) with further coating with Fe^3+^-polyphenol network (MPN) provided reliable (99.7%) destruction of methicillin-resistant bacteria *S. aureus* and *P. aeruginosa* in a murine abscess model [89]. Another MOF (ZIF8/Au-GOx) based on Au and GOx suppressed the growth of bacteria *S. aureus* and *E. coli* at very low concentrations (4 μg/mL and 8 μg/mL, respectively) [90].

The healing of wounds in diabetes is a big problem. Cu^2+^ ions have been shown to stimulate angiogenesis to improve the healing of chronic wounds, and copper oxide nanoparticles exhibit antibacterial activity. In this regard, MOF samples (HvCuO@GOx) were developed to effectively accelerate the healing of diabetic wounds with GOx and hybrid virus-like hollow mesoporous CuO nanoparticles [91]. In the case of local hyperglycemia in bacterial biofilms, MOF catalyzed the formation of H_2_O_2_ with the generation of toxic OH^−^, and then Cu^2+^ caused the proliferation and keratinocytes’ migration. More importantly, after the destruction of the bacteria in the inflammatory phase of wound healing, CuO nanoparticles acted as catalase-mimicking agents to produce large amounts of O_2_ to alleviate hypoxia by catalyzing intracellular H_2_O_2_.

Here, we should recall another development, which is antibacterial nanocolors (MNP-GOxNF) functionalized with amine (NH_2_-MNP) and containing GOx, as well as Cu nanoparticles [95]. The combination of GOx and Cu seems to be very successful for creating highly effective antimicrobials with a long period of their productive and effective use.

An interesting result was obtained with a strong synergistic antimicrobial effect, which is predetermined by the components of a new MOF, which contained lysozyme in the outer layer, an NH_2_-MIL-88B(Fe) shell with carvacrol and Fe_3_O_4_@polyvinylpyrrolidone nanoparticles [94]. These MOF samples had the capability of controlled release of carvacrol. This original type of MOF allowed bacterial cells to be captured by electrostatic attraction, and then “MOF-bacteria” aggregates could be assembled using a magnet. Lysozyme in MOF catalyzed the hydrolysis of peptidoglycan in bacterial cell walls, and carvacrol released from MOF destroyed the bacterial cell membrane under the action of near-infrared radiation. The developed MOF samples at a concentration of 100 µg/mL caused 100% death of both *E. coli* and *S. aureus*, when cell concentration was 10^6^ CFU/mL. Since the MOF possessed low cytotoxicity in vitro, so potentially it can be used in the biomedical and food industries, as well as in ecology.

Thus, by joining enzymes with MOF, it is possible to obtain various new multifunctional materials with antibacterial properties, but today preferences among such enzymes are given to GOx. Today it is well known that, in principle, MOF with enzymes can be combined with various commonly used materials (gels, fibers, composites, etc.) [96], then in the near future we should expect the appearance of new functionalized materials with antimicrobial properties, possibly based on enzymes other than GOx.

## 4. Conclusions

Thus, it is possible to bring highly effective antibacterial properties to materials that may be synthetic, natural, fibrous and solid biocompatible materials, as well as metal–organic frames via their functionalization by certain enzymes. In most cases, hydrolases and oxidoreductases are used to obtain antibacterial materials. Some enzymes, such as lysozyme and glucose oxidase, are effective as independent antimicrobial agents; however, combinations of enzymes (proteases, carbohydrases, etc.) can act more effectively against a wide range of pathogenic microorganisms. In addition, enzymes can be used in combination with metal nanoparticles, antibiotics and antimicrobial peptides to enhance their synergistic effect against pathogenic microorganisms. A variety of enzymes and their targeted antimicrobial action in the composition of functionalized materials enable growth decrease in yeast cells and all types of bacterial pathogens, as well as the destruction or prevention of the formation of biofilms. The immobilization of enzymes in such materials can significantly increase their stability and functional efficiency. The obtained materials, especially those intended for medical purposes, are tested for cytotoxicity and usually characterized by their low level or their complete absence. The materials can be used to solve different tasks in various fields of human activity and needs. Recently developed biocomposite materials are described by high antibacterial properties and have a notable potential for their use in the production of various implants and medical textiles. Current achievements in the field of enzyme engineering and computer modeling form the basis for the development of more effective antimicrobial enzymes and their application. Thereby, new multifunctional materials with combined antimicrobial action have the potential for further development and application, especially due to the epidemic situation in the world and the growing number of antibiotic-resistant pathogens. 

## Figures and Tables

**Table 3:** Antimicrobial MOF functionalized by enzymes.

MOF [Reference]	Antibacterial Properties	Effect of Action
MIL@GOx-MIL NRs (NH_2_-BDC and FeCl_3_·6H_2_O) [83]	5μg/mL MIL@GOx-MIL inhibits *S. aureus* growth. No biofilms formation was revealed at 80 µg/mL	The enzyme catalyzed conversion of glucose to gluconic acid reducing pH from 7.4 to 4 and MIL NRs continually produced H_2_O_2_ and toxic hydroxyl radicals (HO-)
GOx in mesoporous CeO_2_ hollow sphere [84]	100 μg/mL MOF efficiently eliminate 99.9% bacteria in the wound tissues (*E. coli* and *S. aureus*)	Production of highly toxic HO- radicals via a cascade catalytic reaction; Gluconic acid decreased the pH value that boosting the peroxidase-like catalytic performance of mesoporous CeO_2_
MOF (GOx@Fe-ZIF-TA) Fe-doped zeolitic imidazolate framework (ZIF) etching by tannic acid [85]	Complete inhibition of *E. coli* growth was observed	Transformation of surplus glucose to gluconic acid and H_2_O_2_, which was transferred by Fe(II) to antibacterial OH^−^ in infected diabetic wounds
MnFe_2_O_4_@MIL/Au&GOx [86]	Inhibition of bacterial growth; MIC values for *E. coli* and *S. aureus* were 125 and 31.2 μg/mL, respectively	Continuous conversion of glucose into gluconic acid and H_2_O_2_. MnFe2O4@MIL/Au demonstrated increased peroxidase (POD)-like activity and catalyzed transformation of H_2_O_2_ to large amounts of toxic-reactive oxygen species
GOx in 2D Cu-TCPP(Fe) [87]	Inhibition of bacterial growth rates were 88% and 90% for *E. coli* and *S. aureus*	Conversion of glucose to gluconic acid and production of H_2_O_2_; pH decreasing and activation of the peroxidase-like activity of 2D Cu-TCPP(Fe) nanosheets with production of toxic OH^−^ radicals
GOx in Cu/Zn bimetal MOF (Zn(NO_3_)_2_ and Cu(NO_3_)_2_ and 2-methylimidazole) [88]	Inhibition of bacterial growth of both *E. coli* and *S. aureus* was up to 90%	Generation of H_2_O_2_ and its further conversion to OH^−^ radicals by the Cu^2^+ ions; that blocks the nutrient/energy supply for bacteria and triggers a Fenton(-like) reaction; glutathione depletion. All these reactions lead to highly efficient bactericidal effect through synergistic starvation/chemodynamic therapy.
ZIF-ICG@ZIF-GOx@MPN (Indocyanine green (ICG) and GOx were incorporated into homologous zeolitic imidazolate framework-8 (ZIF-8) nanoparticles coating with metal polyphenol network (MPN) composed by Fe^3+^ and tannic acid [89]	Killing of bacteria *S. aureus* and *P*.*aeruginosa* was with efficiency up to 99.7%	Robust OH^−^ radical generation in combination with O_2_ under irradiation induce oxidative damage of pathogenic bacteria
ZIF8/Au-GOx (2-methylimidazole and Zn(NO_3_)_2_·-ZIF8) [90]	Inhibition of bacterial growth; MIC was 4 μg/mL for *S. aureus* and 8 μg/mL for *E. coli*	Generation of ROSand gluconic acid
GOx HvCuO@GOx (hybrid hollow virus-like mesoporous CuO nanospheres) [91]	Inhibition of bacterial growth of *S. aureus*. and *E. coli;* HSHvCuO@GOx dressing decreased amounts of bacterial cells down to 11.5% and 3.3% for 9 and 15 days during wound healing	HvCuO@GOx nanospheres can be efficiently adhered on bacterial surfaces and then activated by the high glucose concentration in biofilm matrix with further generation of toxic OH^−^ radicals and release of Cu^2+^
GOx with L-arginine in CuBDC (L-Arg/GO*x*@CuBDC) [92]	Fenton-like catalytic activity with production of toxic radicals ONOO^−^ and NO	Inactivation of bacterial growth of *E. coli* and *S. aureus* was 97% at 38 μg/mL and 3.8 μg/mL, respectively
GOx with L-arginine in mesoporous Fe_3_O_4_@SiO_2_ [93]	80 μg/mL MOF with GOx and L-arginine can reduce amounts of bacterial cells (*S. aureus*. and *E. coli*) in 1000-100 000 times	Generation of H_2_O_2_ and gluconic acid from glucose
Fe_3_O_4_@PVP@MIL-88B(Fe)–NH- lysozyme/carvacrol Polyvinylpyrrolidone (PVP) [94]	100 μg/mL MOF can cause 100% inhibition of bacterial growth of *E. coli* and *S. aureus* when cell concentration was 10^6^ CFU/mL	The lysozyme degrades the peptidoglycan on bacterial cell wall and carvacrol damages the cell membrane under near-infrared irradiation
